# Design and Calibration of an Instrumented Seat Post to Measure Sitting Loads While Cycling

**DOI:** 10.3390/s20051384

**Published:** 2020-03-03

**Authors:** Dieltiens Sien, D’hondt Jordi, Marc Juwet, Keivan Shariatmadar, Mark Versteyhe

**Affiliations:** 1 KU Leuven Technology Campus Ghent, 9000 Gent, Belgium; jordi.dhondt@kuleuven.be (D.J.); marc.juwet@kuleuven.be (M.J.); 2 KU Leuven Campus Bruges, 8000 Bruges, Belgium; keivan.shariatmadar@kuleuven.be (K.S.); mark.versteyhe@kuleuven.be (M.V.)

**Keywords:** instrumented seat post, cycling loads, six-component sensor, strain gauge based

## Abstract

Traditional instrumented seat posts determine context-induced seat loads to analyze damping properties. This paper presents an enhanced instrumented seat post able to measure all six load components to resolve user-induced seat loads. User-induced cycling loads consist of all loads the user applies to the bicycle during cycling and is measured at the steer stem, the seat post, and the pedals. Seat loads are essentially uncharted territory, as most studies only address pedal loading to study cycling technique. In this paper, a conventional seat post is redesigned by equipping it with a u-shaped component and strain gauges. The instrumented seat post is straightforward thanks to (i) the simple design, (ii) the gravitational calibration method, and (iii) the permitted clearance on the strain gauge alignment. Analyzing mean seat loading in function of the pedal cycle can provide extra insights into cycling technique and the related injuries. It is an interesting addition to the universally adopted method of utilizing singular pedal loads.

## 1. Introduction

Initial instrumented seat posts are developed to analyze the damping characteristics of different bicycle configurations based on the amount of energy that is exchanged between the vibrating bicycle and the cyclist [1,2] Loading due to unsmooth pavement (context-induced cycling loads) and accelerations are measured in the normal and the anterior directions, and the absorbed power is calculated to analyze the level of comfort [3].

**Figure 1:**
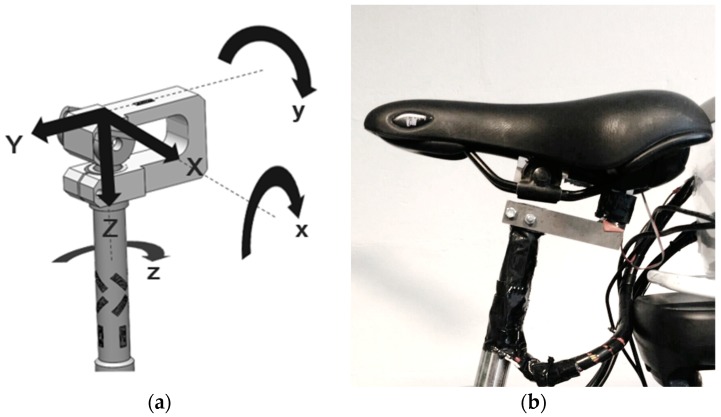
From left to right: (**a**) measured loads for which X = lateral force; Y = anterior force; Z = normal force; x = sagittal torque; y= frontal torque; z = transversal torque, (**b**) installed seat post.

Cycling sore related to the seat is the most frequently reported discomfort amongst cyclists [4,5]. Skin injuries of the groin (e.g., ulceration, bruising, chafing, boils) and soreness of the ischial tuberosities and the pelvis are frequently reported by recreation cyclists [4,6]. Normal forces form the primary cause of a reduced arterial blood flow in the skin and the related skin injuries. Though, according to Bennet et al., the magnitude of the normal forces required to impede the blood flow is halved when the skin is simultaneously loaded in shear [7]. Additionally, other researchers point out the necessity of shear loading for skin tissue breakdown during cycling [4,8,9]. For this reason, analyzing context-induced sitting loads is not sufficient to analyze the sitting comfort while cycling. This paper presents an instrumented seat post to measure all user induced sitting loads, i.e., the anterior, the normal, and the lateral forces as well as the sagittal, the frontal, and the transversal torques, as is presented in Figure 1.

Seat loading is to a great extent dependent of the cyclist’s technique. Recreational cyclists present a great variability in cycling technique due to kinematic differences and the differences in muscle activity [10,11]. Competitive cyclists are trained to adapt the most efficient cycling technique and posture; they present a reduced variability predominantly caused by differences in muscle recruitment [10]. To average out the within-subject variability, loads are expressed in function of one pedal cycle, and mean load patterns are determined. The load patterns are of interest to analyze cycling techniques related to cycling sores and the influence of external factors such as pedal power assistance. Knowledge of favorable and unfavorable load patterns and the influence of bicycle type or posture can decrease the risk of injuries.

Unlike straightforward acceleration measurements with commercial accelerometers, ready-made load cells are difficult to integrate in a conventional seat post because (i) there is only limited space available between the cyclist’s inner thighs, and (ii) the amplitude of the loads vary drastically in the three dimensions, e.g., the normal force is ten times higher than the lateral one. In the past, several unique seat post sensors were designed, predominantly based on cantilever beam principles, utilizing strain gauges to measure material stress. In this work, we observe that they all show some shortcomings: (i) they measure only two dimensional loads to calculate the absorbed power [1], they measure the vertical force directly on the seat post, which results in a very low signal-to-noise ratio [12], (ii) they assume that the point of application of the loads on the seat is fixed and use independent pairs of half bridges to determine the forces [13], and (iii) they need expensive precision equipment to calibrate the sensor dynamically due to the high frequency vibrations [14]. A straight-forward instrumented seat post is developed herein that measures all user-induced loads during in-situ cycling and does not compromise the aforementioned shortcomings.

The seat post is part of a fully instrumented bicycle utilized to determine the user-induced loads on all areas of contact between cyclist and bicycle. Unique load cells are implemented in steer, seat, and pedals, and an encoder is added to the bottom bracket. The load cells are all expressed in the same reference frame to estimate forces and torques near the joints of the cyclist; reactive forces and torques measured by the load cells are transferred through the body segments as a bar linkage system. The loading is expressed in mean loading patterns over one pedal cycle, and the influence of electrical pedal power assistance and posture are analyzed on recreational bicycles for women. 

## 2. Materials and Methods

A straightforward seat post is developed herein to analyze cycling characteristics. The research addresses the construction method of the seat post and the data processing to create graphs from which cycling characteristics can be derived. 

### 2.1. Seat Post Design

The seat post is considered a cantilever beam that deforms elastically under a certain region of tension in function of the mechanical characteristics. The elastic deformation is directly proportional to the acting loads. Strain gauges are attached on the deforming material, causing a change of inner resistance and creating an output signal from which the acting load can be derived, which is given by (1).

(1) $$є=\frac{Mc}{IE}$$

where є is the strain, *M* is the moment, *c* is the distance from the center of the beam to the point where the strain is being measured, *E* is the modulus of elasticity, and *I* is the moment of inertia. 

The diameter of the seat post is 27 mm, standard for a conventional city bike. The thickness is determined by a stress simulation with a finite element analysis under the seat post loading of a cyclist of 100 kg. Table 1 presents the forces acting on a seat post for a cyclist of 100 kg [15].

The seat post should easily withstand extreme seat loads and prevent plastic deformations. The accuracy needs to be maximized; the elastic deformation needs to be high enough and have the same order of magnitude for every direction. For a standard seat post, 2 mm of thickness is ideal for the lateral and the anterior forces. The forces are considerably limited but cause significant elastic deformations due to the leverage effect of the seat post. On the contrary, normal forces are difficult to measure on the seat post. They cause a minimal elastic deformation, and the signal-to-noise ratio is too low. Enlarging the voltage and the strain-gauge resistance for thermal dissipation can enhance the signal [2], though creating an extra zone that elastically deforms with the same order of magnitude as the loads in the other directions is preferred [1]. A unique U-shaped component is developed from the structural steel grade S355JR, with the specific standard EN10025-2. It is mounted between the seat and the seat post to create a zone exposed to shear forces that elastically deforms under the normal force without interfering with the system dynamics, as the seat of a recreational bicycle contains springs and extra cushion for damping. The sensitivity is increased without compromising the bandwidth. The minimal distance between the vertical tube of the bicycle frame and the seat measures 12 cm. The instrumented seat post is presented in Figure 1. 

The strain gauge’s resistance change due to deformation is limited. The signal-to-noise ratio is improved by connecting two or four strain gauges with each other in a Wheatstone bridge configuration.

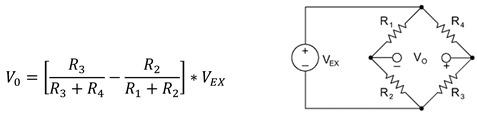
(2)
where *V*_0_ is the output voltage, *V_EX_* is the supply voltage, R1 and R3 are strain gauges attached to the zones that endure tensile stress due to the acting force, and R2 and R4 are attached to the zones that endure compressive stress due to the acting force to maximize the output signal, which is given by (2).

**Figure 2:**
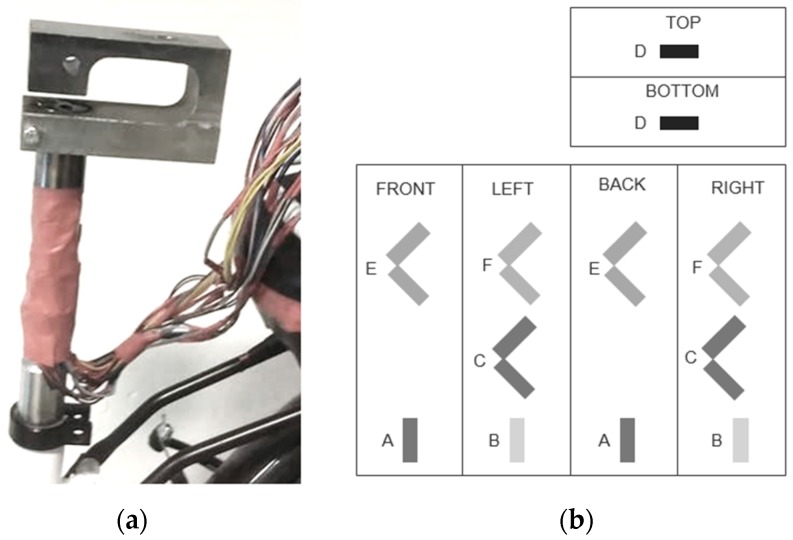
(**a**) the instrumented seat post, (**b**) schema of different views of the seat post to present strain gauge placement for which configuration A: strain gauges to measure sagittal torque; B: strain gauges to measure frontal torque; C: strain gauges to measure transversal torque; D: strain gauges to measure normal force; E: strain gauges to measure anterior force; and F: strain gauges to measure lateral force.

The torques in the sagittal and the frontal plane are measured by placing two strain gauges in a half bridge configuration and attaching them, respectively, on the front-and-back side and the left-and-right side of the seat post (combining R1 with R2). The normal force is likewise measured with a similar half bridge configuration on the top part of the U-shaped component. The lateral force, the anterior force, and the torque in the transversal plane are measured by placing four strain gauges in a full bridge and attaching them on the seat stem in a rosette arrangement. Therefore, a shifting point of application of the seat loading has no effect on the accuracy of the measured forces. A schematic overview is provided by Figure 2.

The resistance of strain gauges is highly dependent on the longitudinal deformation but also presents a minimal change of resistance due to transverse deformation. Alignment errors of the strain gauge placement makes this phenomenon more pronounced. A calibration matrix is deduced that takes the output signals of all strain gauge configurations into account to cancel out this cross-sensitivity, which is given by (3).

(3) $$\left[\begin{array}{ccc} \begin{array}{cc} C_{11} & C_{12}\\ C_{21} & C_{22} \end{array} & \ldots & \begin{array}{c} C_{61}\\ C_{62} \end{array}\\ \vdots & \ddots & \vdots \\ \begin{array}{cc} C_{61} & C_{62} \end{array} & \ldots & C_{66} \end{array}]*[\begin{array}{c} m_{1}\\ m_{2}\\ \vdots \\ m_{6} \end{array}]=[\begin{array}{c} F_{X}\\ F_{Y}\\ F_{Z}\\ M_{X}\\ M_{Y}\\ M_{Z} \end{array}\right]$$

where *C*_11_, …, *C*_66_ are calibration matrix values, *m*_1_, …, *m*_6_ are the measured output signals and *F_x_*, …, *M_z_* are the known forces and torques.

Formula (3) is transformed to formula (4) to determine the calibration matrix:

(4) $$\begin{array}{c} \left[\begin{array}{ccc} \begin{array}{cc} C_{11} & C_{12}\\ C_{21} & C_{22} \end{array} & \ldots & \begin{array}{c} C_{61}\\ C_{62} \end{array}\\ \vdots & \ddots & \vdots \\ \begin{array}{cc} C_{61} & C_{62} \end{array} & \ldots & C_{66} \end{array}\right]\\ \;={\left([\begin{array}{ccc} \begin{array}{cc} m_{Fx1} & m_{Fx2}\\ m_{Fy1} & m_{Fy2} \end{array} & \ldots & \begin{array}{c} m_{Fx100}\\ m_{Fy100} \end{array}\\ \vdots & \ddots & \vdots \\ \begin{array}{cc} m_{Mz1} & m_{Mz2} \end{array} & \ldots & m_{Mz100} \end{array}]*{\left[\begin{array}{ccc} \begin{array}{cc} m_{Fx1} & m_{Fx2}\\ m_{Fy1} & m_{Fy2} \end{array} & \ldots & \begin{array}{c} m_{Fx100}\\ m_{Fy100} \end{array}\\ \vdots & \ddots & \vdots \\ \begin{array}{cc} m_{Mz1} & m_{Mz2} \end{array} & \ldots & m_{Mz100} \end{array}\right]}^{T}\right)}^{-1}\\ \;*{\left[\begin{array}{ccc} \begin{array}{cc} m_{Fx1} & m_{Fx2}\\ m_{Fy1} & m_{Fy2} \end{array} & \ldots & \begin{array}{c} m_{Fx100}\\ m_{Fy100} \end{array}\\ \vdots & \ddots & \vdots \\ \begin{array}{cc} m_{Mz1} & m_{Mz2} \end{array} & \ldots & m_{Mz100} \end{array}\right]}^{T}*[\begin{array}{ccc} \begin{array}{cc} F_{x1} & F_{x2}\\ F_{y1} & F_{y2} \end{array} & \ldots & \begin{array}{c} F_{x100}\\ F_{y100} \end{array}\\ \vdots & \ddots & \vdots \\ \begin{array}{cc} M_{z1} & M_{z2} \end{array} & \ldots & M_{z1oo} \end{array}] \end{array}$$

The measured output signals (*m*_1_, …, *m*_6_) can vary when loading is applied at high frequencies. Seat posts that measure context induced loads focus on vibrations and need to be calibrated dynamically, as the sensor structural dynamics can cause amplification at resonances and physical attenuations above its natural frequencies [1,2]. This seat post measures the substantially slower user-induced loads. As a recreational cyclist cycles at 50 rpm (0.8 Hz) and a competitive one at 100 rpm (1.6 Hz), and the pedal cycle exists of four phases—set up, down stroke, pull back, and lift up—the frequency bandwidth of interest is less than 10 Hz. A hammer excitation test is conducted to verify the need of dynamic calibration; if the frequency bandwidth of interest is lower than the natural frequency of the instrumented seat post under test circumstances, a dynamic calibration is superfluous. The strain gauge measurement equipment is limited to a sampling frequency of 100 Hz, and a piezoelectric accelerometer with a higher frequency range is elected. The accelerometer 353B31 (PCB) is mounted on the seat post with a sensitivity of 50 mV/g, a frequency range of 1 to 5000 Hz, and a weight of 20 mg. The sensitivity roll off of the sensor below 1 Hz is typically very smooth. If there was to be a resonance peak below 1 Hz, which is very unlikely, it would be observed it in the measurement.

**Figure 3:**
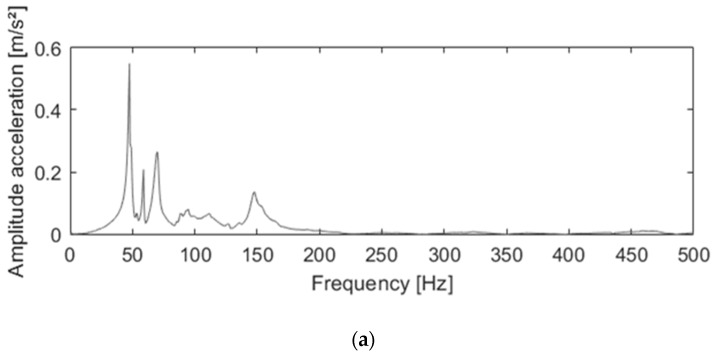

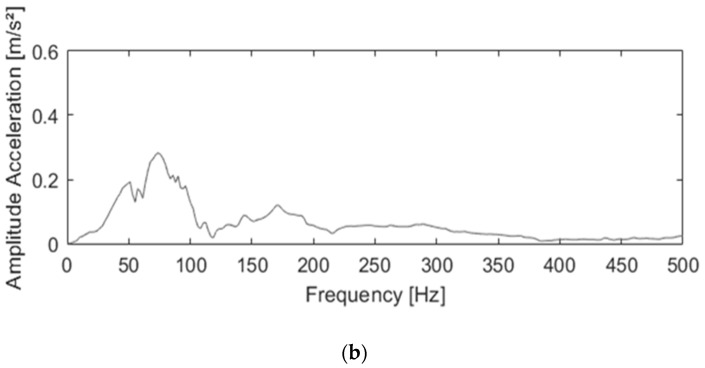
Frequency response in the vertical direction without a cyclist (**a**) and with a cyclist (**b**).

A hammer blow is applied on the seat, and the accelerations in the three dimensions are measured. The test is repeated (i) for a hammer blow in all three the directions and (ii) with and without a test subject of 70 Kg. Figure 3 addresses the frequency response that comes the closest to the frequency bandwidth of interest, namely in the vertical direction. As seen in the figure, the addition of the cyclist has a significant influence on the damping properties, though it has a minimal influence on the natural frequency. When a cyclist sits on the seat post, the natural frequency enlarges in the normal direction from 48 Hz to 51 Hz, in the lateral direction from 59 Hz to 86 Hz, and in the anterior direction from 72 Hz to 76 Hz. Therefore, a heavier cyclist has a positive influence on the natural frequency. The natural frequencies with and without a cyclist fall significantly outside the frequency bandwidth of interest. The data of the experiments are filtered with a cut of frequency of 10 Hz, and a dynamic calibration is superfluous.

**Figure 4:**
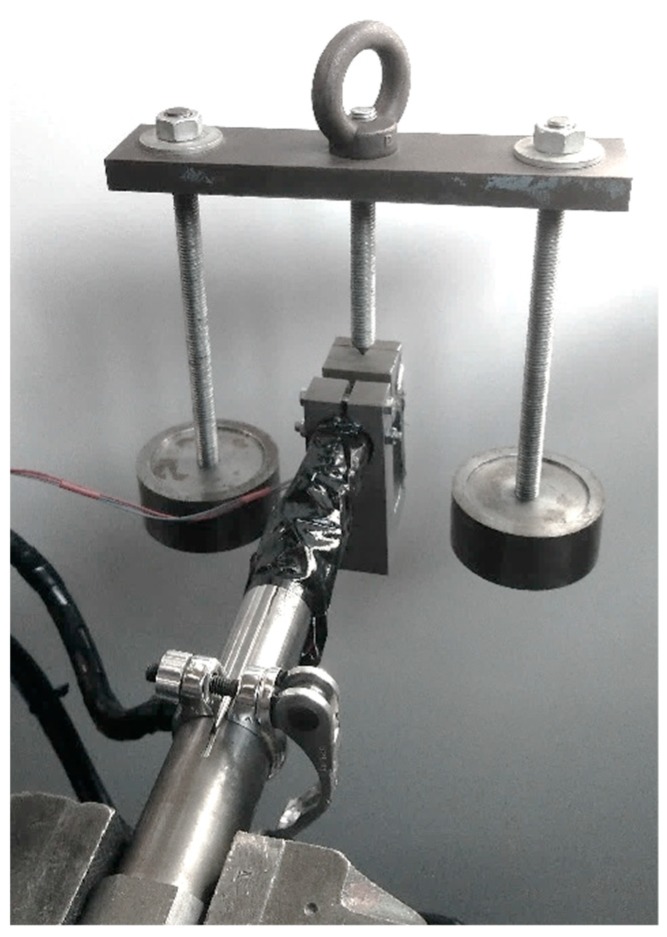
Setup example to relate sensor output to a known force torque combination.

The test demonstrates that a static calibration is sufficient; multiple weights are applied on the seat post on various lever lengths, each time resulting in a known combination of a force with one or two torques, and the sensor output is measured. As seen in formula (4), the test is repeated one hundred times with a different load, orientation, and point of application. The results are subtracted from equal measurements without loading. The loading range reaches up to the forces acting on a seat post for a cyclist of 100 kg, as seen in Table 1. Figure 4 is an example of one of the setups.

The standard error percentage of full scale is determined to quantify the variation between the calculated and the measured forces relative to the maximal loads acting on the seat post during the calibration (5). The maximal error percentage of full scale is furthermore calculated to determine the maximal error found between the calculated and the measured values relative to the maximal loads acting on the seat post during the calibration (6).

(5) std.error % FS=∑i=1N(mFi−Fi)N−fFS100

(6) $$max.error\;\%\;FS=\frac{Max|m_{Fi}-F_{i}|}{FS}100$$

where *N* is the number of samples, *f* is the degrees of freedom of the calibration matrix, and *FS* is the maximal load value utilized to calibrate the sensor (as seen in Table 1). 

Both parameters are widely used in literature to define the accuracy of a sensor [16,17] though, to our knowledge, they are missing in literature addressing seat post sensors. 

### 2.2. Data Processing

The raw data are processed in order to make it easily interpretable for bike fitters; mean load graphs are constructed, which depicts the typical cycling technique of the test subject. The magnitude and the direction of the cycling loads are highly dependent of the phase in the pedal cycle; therefore, mean unidirectional loads are expressed in function of one pedal cycle, starting when the right pedal is in his upper dead point. An absolute encoder is inserted in the bottom crank to measure the pedal arm angle. The encoder consists of a toothed disk clamped over the bottom crank and an optical sensor fixated on the frame. 

The sensor counts the gaps, starting from a wider reference gap, and the pedal arm angle is calculated. Poor data caused by pedaling backwards, no pedaling, or pedaling unsteadily are removed. Only when a minimum of five consecutive pedal cycles are counted with a deviation of less than 5% are the data saved. The results are filtered with a low-pass zero phase shift Butterworth filter (3, 0.1,”low”) and plotted in function of the phase in the pedal cycle.

### 2.3. Testing

The load cell is tested on a women’s city-bike during a relaxed cycling tour of 15 min on a straight lane next to a river. The maximal seat to pedal distance is set as 108% of the inner leg length, and the cyclist weights 60 kg. 

## 3. Results

### 3.1. Output Accuracy

Table 2 addresses the calculated accuracy of the calibration matrix.

The standard error percentage of full scale is the highest for the frontal torque (3.02%), followed by the lateral force (1.14%), the transversal torque (0.58%), the normal force (0.34%), the sagittal torque (0.28%), and the anterior force (0.15%). The maximal error percentage of full scale is the highest for the normal force (1.56%), followed by the frontal torque (1.43%), the sagittal torque (0.51%), the lateral force (0.46%), the anterior force (0.37%), and the transversal torque (0.25%).

### 3.2. Output Results

**Figure 5:**
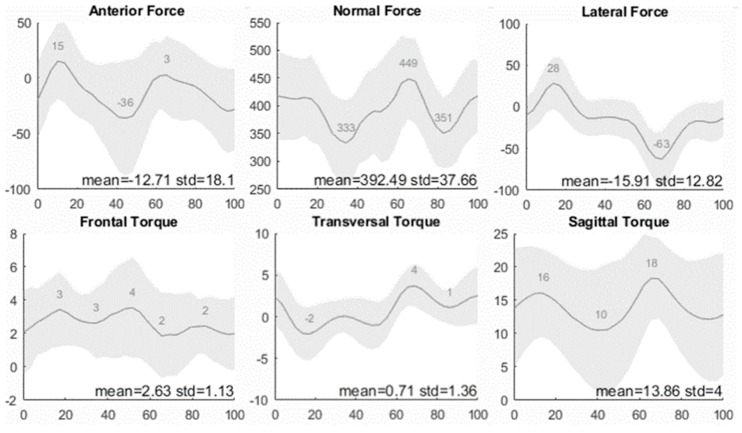
Seat forces (N) and torques (Nm) expressed in function the pedal cycle (%).

Figure 5 presents the output results of the cycling tour. The cyclist produced a mean power output of 70 W and a mean pedaling cadence of 50 rpm. Erroneous data due to disturbed pedaling are deleted, and 689 cycles remain.

The normal axis represents the seat load expressed in Newton, and the horizontal axis represents the phase of the pedal cycle, wherein the right pedal starts (0%) and ends (100%) in the upper normal position (dead center). The grey area represents the variation. The Anderson–Darling Test for a Normal Distribution fails to reject the null hypothesis, which states that the data comes from a normal distribution at a 5% significance level; therefore, it can be concluded that 95% of the data are situated in the gray area.

The mean force in the anterior direction is directed posteriorly and has a value of 13 N with a standard deviation of 18 N. The pattern knows two distinctive cycles with an anteriorly directed peak in the first and the third quartile of the pedal cycle and a posteriorly directed peak in the second and the fourth quartile. The anteriorly directed peaks are located at 17% and 67% with a value of 13 N and 3 N, respectively. The two posteriorly directed peaks are located at 43% and 93% with values of 36 N and 30 N, respectively. The normal mean force is 392 N with a standard deviation of 38 N. Two distinctive cycles are considered with maxima at 3% and 67% of 418 N and 449 N, respectively. The minima are located at 37% and 83% with values of 333 N and 351 N, respectively. The lateral mean force is 16 N directed to the left with a standard deviation of 13 N. There are two distinctive cycles with a peak of 28 N directed to the left at 17% and a peak of 63 N directed to the right at 70%. The frontal torque has a mean value of 3 N with a standard deviation of 1 N. The mean transversal torque is 1 N with a standard deviation of 1 N. Two oppositely directed cycles are distinguished. The first is directed to the right and has two peaks at 10% and 50% with values of 2 N and 1 N, respectively. The second is directed to the left and has two peaks at 70% and 100% with values of 4 N and 3 N, respectively. The sagittal mean torque is 14 N with a standard deviation of 4 N. The pattern knows two distinctive cycles with maxima at 17% and 67% of 16 N and 18 N, respectively. The minima are located at 43% and 93% with values of 11 N and 12 N, respectively.

## 4. Discussion

### 4.1. Output Accuracy

To our knowledge, the accuracy of sensors implemented in seat posts are not described in literature, and the calibration methodology is not standardized. Research addressing sensors implemented in pedals presents a maximal error percentage of full scale and a standard error of full scale around 3% [16,17]. In our research, the standard error percentage of full scale is less than 1% for the anterior force, the normal force, the transversal torque, and the sagittal torque, which is three times lesser. The standard error for the lateral force is 1.14% and for the frontal torque is 3%. The maximal error percentage of full scale is less than 1% for the anterior force, the lateral force, the transversal torque, and the sagittal torque. The maximal error of the normal force for the normal force is 1.55% and for the frontal torque is 1.43%. Compared with the results of literature, the sensor performs significantly better. A higher accuracy can be obtained by calibration with a three-dimensional tensile tester and with an increased amount of samples, though this influences the costs drastically [18,19,20].

### 4.2. Output Results

The mean measured normal force covers 65% of the cyclist’s weight, the mean posterior force covers 2%, and the mean lateral force covers 3%. In literature, the percentages are in the same range, respectively, 49–52%, 11–12%, and 4–5% for racing bicycles with a power output of 125 Watt [5]. The higher percentage of the normal seat force could be explained by the lower power output to the pedals and the related higher loading on the seat post. The smaller percentage of the posterior force could be explained by the more upright posture on a city bike versus a racing bike. 

The cycling technique is easily interpretable from the graphs; all loads except the frontal torque have two symmetric cycles based on the thigh tilt due to the right pedal movement and the left pedal movement. The first half of the pedal cycle represents the power phase of the right pedal and the recovery phase of the left pedal, and the second half represents the power phase of the left pedal and the recovery phase of the right pedal. The transversal torque and the lateral force have an opposite direction in the power phase of the right pedal versus the power phase of the left pedal. When the right pedal enters the first quartile of the pedal cycle, all loads peak except the frontal torque. In the first quartile, the right pedal is in its power phase, directing the lateral force to the left and the transversal torque counterclockwise. The force is transferred from the right thigh to the right pedal to maximize pedal power, suggesting a lateral tilt of the hip and an exorotation of the torso. When the pedal enters the second quartile, the thigh tilt is decreased, and load minima are reached for the sagittal torque and the forces, though transversal torque decreases are marginal. This suggests that the torso is still rotated. After the lower dead center, the left pedal takes over, and the process of the first and the second quartile repeats itself, though with the lateral force, a transversal torque is directed oppositely. The frontal torque does not present a clear pattern; nevertheless, the peaks of the standard deviation point upwards in the first half of the pedal cycle and downwards is the second half, supporting the aforementioned hypothesis about the lateral tilt of the hips.

The shape of the wide recreational saddle utilized in the tests makes room for a large range of motion and different postures. The absence of clipless pedals enables various types of ankle movement, and the relaxed upright posture does not demand folded nor locked elbows. Consequently, the test setup empowers a high variability in kinematics and muscle recruitment.

There is a significant difference in the maximal normal force produced in the power phase of the left pedal (449 N) versus the right (418 N). The same pattern presents itself in the lateral force (with 63 N versus 28 N, respectively), and in the posterior force (with 36 N versus 30 N, respectively). This can have multiple causes: (i) the right leg is dominant and produces a higher pedal power than the left, (ii) the subject is not sitting straight on the seat, or (iii) the subject has an unsymmetrical body built (e.g., scoliosis or rotated pelvis). An asymmetrical cycling technique could lead to an overdevelopment of glutes and hamstrings of the dominant leg and cycling sores around the ischial tuberosities of the opposite side. Statistical research of seat post data could lead to various new insights to tackle maladaptive cycling techniques, such as muscle imbalance and seat sore, and define the perfect fit. The principle of the sensor could be used in other applications and areas (robot arms, motorcycle, wheelchair, etc.) to measure the six degrees of freedom loading with straightforward and better accuracy.

## 5. Conclusions

A straightforward instrumented seat post sensor is developed that measures all six components of the user-induced loads during in-situ cycling on conventional bicycles, and a method is presented to easily interpret the data. The sensor has a maximal standard mean error of 3% and is not affected by a shifting point of application of the cyclists’ backside. The new sensor is straightforward to construct, to calibrate, and to implement on a standard bicycle and is therefore commercially interesting. The instrumented seat post is a meaningful extension on the widely utilized instrumented pedals to determine cycling technique. The instrumented seat post forms part of a fully instrumented bicycle to determine all the user induced cycling loads, derive the joint loading, and analyze the influence of external parameters (e.g., pedal assistance, cycling duration, bicycle type) and user characteristics on cycling technique.

## Figures and Tables

**Table 1:** Seat post loading during cycling.

	Lateral (X)	Anterior (Y)	Normal (Z)
Percentage of body weight applied to the seat post	4–5%	11–12%	49–52%
Seat post loading for a cyclist of 100 kg	40–50 N	110–120 N	490–520 N

**Table 2:** Seat post loading during cycling.

Load	Std. Error Percentage Full Scale (FS)	Max. Error Percentage FS
Anterior force	0.15	0.37
Normal force	0.34	1.55
Lateral force	1.14	0.46
Frontal torque	3.02	1.43
Transversal torque	0.58	0.25
Sagittal torque	0.28	0.51
